# VEGF (Vascular Endothelial Growth Factor) Induces NRP1 (Neuropilin-1) Cleavage via ADAMs (a Disintegrin and Metalloproteinase) 9 and 10 to Generate Novel Carboxy-Terminal NRP1 Fragments That Regulate Angiogenic Signaling

**DOI:** 10.1161/ATVBAHA.118.311118

**Published:** 2018-06-07

**Authors:** Vedanta Mehta, Laura Fields, Ian M. Evans, Maiko Yamaji, Caroline Pellet-Many, Timothy Jones, Marwa Mahmoud, Ian Zachary

**Affiliations:** From the Centre for Cardiovascular Biology and Medicine, Division of Medicine, The Rayne Building, University College London, United Kingdom.

**Keywords:** cytokines, endothelial cells, phosphorylation, proteolysis, signal transduction

## Abstract

**Objective—:**

NRP1(neuropilin-1) acts as a coreceptor for VEGF (vascular endothelial growth factor) with an essential role in angiogenesis. Recent findings suggest that posttranslational proteolytic cleavage of VEGF receptors may be an important mechanism for regulating angiogenesis, but the role of NRP1 proteolysis and the NRP1 species generated by cleavage in endothelial cells is not known. Here, we characterize NRP1 proteolytic cleavage in endothelial cells, determine the mechanism, and investigate the role of NRP1 cleavage in regulation of endothelial cell function.

**Approach and Results—:**

NRP1 species comprising the carboxy (C)-terminal and transmembrane NRP1 domains but lacking the ligand-binding A and B regions are constitutively expressed in endothelial cells. Generation of these C-terminal domain NRP1 proteins is upregulated by phorbol ester and Ca^2+^ ionophore, and reduced by pharmacological inhibition of metalloproteinases, by small interfering RNA-mediated knockdown of 2 members of ADAM (a disintegrin and metalloproteinase) family, ADAMs 9 and 10, and by a specific ADAM10 inhibitor. Furthermore, VEGF upregulates expression of these NRP1 species in an ADAM9/10-dependent manner. Transduction of endothelial cells with adenoviral constructs expressing NRP1 C-terminal domain fragments inhibited VEGF-induced phosphorylation of VEGFR2 (VEGF receptor tyrosine kinase)/KDR (kinase domain insert receptor) and decreased VEGF-stimulated endothelial cell motility and angiogenesis in coculture and aortic ring sprouting assays.

**Conclusions—:**

These findings identify novel NRP1 species in endothelial cells and demonstrate that regulation of NRP1 proteolysis via ADAMs 9 and 10 is a new regulatory pathway able to modulate VEGF angiogenic signaling.

## Abstract 2

Supplemental Digital Content is available in the text.

NRP1 (neuropilin-1) is a transmembrane glycoprotein that is required for embryonic neuronal and vascular development.^1,2^ In endothelial cells, NRP1 acts as a coreceptor for VEGF (vascular endothelial growth factor)-A, forming heterocomplexes with the VEGFR2 (VEGF receptor tyrosine kinase), which mediate optimal VEGF signaling and endothelial cell migration essential for angiogenesis.^3–5^ NRP1 also mediates endothelial signaling pathways essential for endothelial cell motility, including tyrosine phosphorylation of p130Cas and paxillin, and activation of small GTPases.^6–11^

**See cover image**

Alternative splicing of mRNA encoding NRP1 gives rise to several sNRP1 (soluble NRP1) isoforms containing the a1/a2 and b1/b2 domains but lacking the MAM (c), transmembrane, and cytoplasmic domains.^3,12^ These species encode isoforms varying from 551 to 704 amino acid residues in size, at least 2 of which, s_12_NRP1 (NRP1 isoform b) and s_IV_NRP1 (NRP1 isoform c), are expressed in protein form. sNRP1s may act as decoys, competitively binding and sequestering VEGF_165_, thereby mimicking the effect of VEGF_165_ withdrawal and negatively regulating angiogenesis in, for example, tumor cell growth.^13^ It has also been proposed that binding of sNRP1 dimers to VEGF_165_ could mediate VEGF delivery to endothelial cell VEGFR2, thereby promoting angiogenesis.^14^ Another, less well-studied, mechanism for generation of NRP1 protein isoforms is posttranslational proteolytic cleavage of the full-length protein. Production of an sNRP1 species was reported to be induced by the Ca^2+^ ionophore, ionomycin, in COS-7 and mouse embryo fibroblasts via ADAM (a disintegrin and metalloproteinase)10-dependent pathway.^15^ Furthermore, NRP1 exodomain shedding in axons via ADAM10 and ADAM17 has been shown to play an important physiological role in mediating axonal desensitization to Sema3A essential for embryonic neuronal homing.^16^ However, the relevance of ADAM-dependent NRP1 cleavage in endothelial cells is unclear. Furthermore, neither the nature nor the function of NRP1 species generated by cleavage is known.

Herein, we report the expression in endothelial cells of novel NRP1 protein species containing the cytoplasmic domain, but lacking the regions of the extracellular domain essential for binding of VEGF and semaphorin ligands. We demonstrate that expression of NRP1 cytoplasmic domain species in endothelial cells is mediated through ADAM-dependent cleavage and is stimulated by VEGF. Overexpressing NRP1 species containing the cytoplasmic domain inhibited VEGF stimulation of receptor signaling, cell migration, and angiogenesis. These findings identify a novel mechanism in endothelial cells that may potentially regulate the VEGF/NRP1 signaling network, with implications for understanding the control of angiogenesis.

## Materials and Methods

The data that support the findings of this study are available from the corresponding author on reasonable request.

### Cells

Human umbilical vein endothelial cells (HUVECs) were purchased from TCS CellWorks (Bucks, United Kingdom) and cultured in endothelial basal medium (Cambrex BioScience Ltd, Nottingham, United Kingdom) supplemented with gentamicin-ampicillin, epidermal growth factor, bovine brain extract (Singlequots; Cambrex), and 10% fetal bovine serum (Life Technologies, Paisley, United Kingdom). HUVECs used in experiments were no more than passage 6. Human coronary artery smooth muscle cells were purchased from PromoCell (Heidelberg, Germany).

### Antibodies, Drugs, and Small Interfering RNAs

Antibodies to the NRP1 carboxy terminus (C-19; sc-7239), glyceraldehyde 3-phosphate dehydrogenase (GAPDH; V-18, sc-20357), ADAM9 (sc-23290), ADAM17 (TACE; C15, sc-6416), and VEGFR2/KDR (kinase domain insert receptor; A-3, sc-6251) were from Santa Cruz Inc (Santa Cruz, CA). NRP1 extracellular domain antibody (catalog no. AF3870) was from R&D Systems (Abingdon, United Kingdom). Phospho-VEGFR2 (Y1175, number 2478) and Notch intracellular cleavage domain (number 4147) antibodies were from Cell Signaling Technology Inc (Danvers, MA). Antibody to ADAM10 was from Sigma Aldrich (catalog no. A2726). Predesigned small interfering RNAs (siRNAs) targeted against ADAMs 9, 10, 17 (Table) or scrambled control were purchased from GE Healthcare (Little Chalfont, United Kingdom) and used for transfection as previously described.^7^ PMA, GI254023X, marimastat, chloroquine, and lactacystin were purchased from Sigma Aldrich (Dorset, United Kingdom); Ionomycin was purchased from Merck Millipore (Herts, United Kingdom).

**Table.**
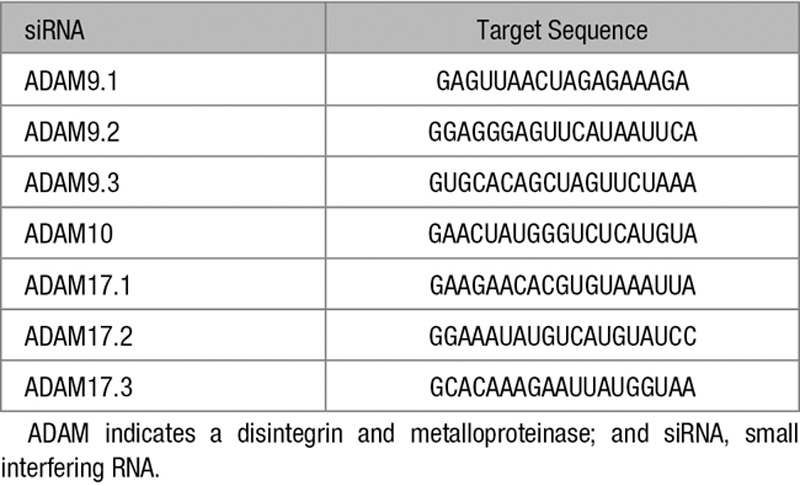
ADAM siRNAs Used in This Study

### Adenovirus Generation

All reagents used for the generation of adenovirus constructs were from Thermo Fisher Scientific. Adenoviruses (Ad) expressing human wild-type NRP1 (Ad.NRP1 WT), NRP1 lacking the intracellular domain (Ad.NRP1ΔC), NRP1 containing only the cytoplasmic and transmembrane domains (Ad.NRP1Cyt-TM), NRP1 containing the cytoplasmic, transmembrane, and juxtamembrane domains (Ad.NRP1Cyt-JM), and NRP1 containing the cytoplasmic, transmembrane, juxtamembrane, and MAM domains (Ad.NRP1Cyt-MAM) were generated using the Gateway system. Briefly, NRP1 open reading frames were subcloned into the pENTR/D-TOPO vector by PCR amplification with primers designed according to the manufacturer’s instructions (listed below) and using directional TOPO cloning. NRP1 adenoviral expression vectors (pAd/CMV/V5-DEST) were generated by recombination, and adenovirus was produced by transfection into host human embryonic kidney 293A cells. Viral particles were released from the human embryonic kidney 293A cells by 3 freeze-thaw cycles and purified using the Adenopure adenovirus purification kit (Puresyn, Inc), the virus titers were then determined using QuickTiterTM Adenovirus Titer quantification kit (Cell Biolabs, Inc), and purified adenoviruses were stored at −80°C. HUVECs were infected with one of the Ads described above or Ad.LacZ at a multiplicity of infection of 100. The primers used for the generation of Ad.NRP1WT, Ad.NRP1ΔC, Ad.NRP1Cyt-TM, Ad.NRP1Cyt-JM, and Ad.NRP1Cyt-MAM were as follows:

Ad.NRP1ΔC:

Forward: 5′-GCTGTCTGTGGGGTCGTGCTGTAGTGTGCCTGTTGGCATAATGG-3′ and,
Reverse: 5′-CCATTATGCCAACAGGCACACTACAGCACGACCCCACAGACAGC-3′
Ad.NRP1WT: Forward: 5′-CACCATGGAGAGGGGGCTGCC-3′,
Ad.NRP1Cyt-TM: Forward: 5′-CACCATGATCCTCATCACCATCATAGCC-3′,
Ad.NRP1Cyt-JM: Forward: 5′-CACCATGGACATTAGTATTAATAACCACATTTCACAA-3′,
Ad.NRP1Cyt-MAM: Forward: 5′-CACCATGACCATACAATCAGAGTTTCCAACA-3′
The reverse primer for the WT, Cyt-TM, Cyt-JM, and Cyt-MAM constructs is: Reverse: 5′-TCATGCCTCCGAATAAGTACTCTGTG-3′.

### Western Blotting

Cytoplasmic and nuclear extracts were obtained using a ProteoJet Cytoplasmic and Nuclear Protein extraction kit (Fermentas Life Sciences, United Kingdom) according to the manufacturer’s instructions. For immunoblotting, cells and cell extracts were prepared by addition of a solution containing 50 mmol/L Tris-HCl (pH 7.5), 1% Triton X-100, 150 mmol/L NaCl, 5 mmol/L EDTA, complete protease inhibitor (Roche; Sussex, United Kingdom), and phosphatase inhibitors I and II (Sigma) and analyzed by sodium dodecyl sulfate-polyacrylamide gel electrophoresis using 4% to 12% Bis-Tris gels (NuPAGE; Life Technologies), followed by electrotransfer on to Invitrolon polyvinylidene difluoride membranes (Life Technologies). Membranes were blocked with 5% (wt/vol) nonfat dry milk and 0.1% (vol/vol) Tween 20 in phosphate-buffered saline for 1 hour at room temperature before being probed with the primary antibody by overnight incubation at 4°C, followed by incubation for 1 hour at room temperature with a horseradish peroxidase-linked secondary antibody (Santa Cruz) and detection using ECL plus reagents (GE Healthcare) in accordance with the manufacturer’s protocol. For immunoblotting of shed NRP1 ectodomain, supernatants from treated cells were pooled together and concentrated using Amicon centrifugal filtration devices with a 10 kDa cut off according to the manufacturer’s instructions, and samples were then prepared for immunoblotting as described above. All immunoblots were quantified by scanning films with a calibration strip and analyzed by densitometry using ImageJ (US National Institutes of Health; http://rsb.info.nih.gov/ij/).

### Co-Immunoprecipitation

HUVECs cultured in 10 cm dishes were infected with the different adenoviral constructs. Forty-eight hours postinfection, the cells were incubated in medium containing 0.5% fetal bovine serum for 16 hours. Next morning, cells were stimulated with 25 ng/mL VEGF for 10 minutes. The cells were then lysed using an IP buffer (PBS containing 0.1% NP-40 with protease inhibitor cocktail). The lysates were immunoprecipitated with an antibody to VEGFR2 (Santa Cruz; sc-6251) using the Dynabeads Protein G Kit (Life Technologies), as per the manufacturer’s instructions. The immunoprecipitated proteins were separated on a 4% to 12% SDS-PAGE gel, transferred to a PVDF membrane and probed with antibodies.

### Sequencing

HUVECs cultured in 15 dishes (15 cm each, VWR International, Leicestershire United Kingdom) were infected with Ad.NRP1. After 48 hours of infection, they were stimulated with Phorbol ester for 24 hours to stimulate the generation of cytoplasmic fragments. Protein lysates were prepared in IP buffer (PBS containing 1% NP-40) and immunoprecipitated with the C-19 antibody using Dynabeads G, as per the manufacturer’s instructions. The immunoprecipitated complex was resolved on a 4% to 12% SDS-PAGE gel, transferred as described above and the PVDF membrane was stained with Ponceau S (Sigma). All positively stained bands <25 kDa were sent for automated N-terminal sequencing by Edman degradation to Alta Biosciences, University of Birmingham.

### Cell Migration Assay

Transwell cell culture inserts (BD Biosciences, Oxford, United Kingdom) were inserted into a 24-well plate. Serum-free medium with or without the indicated growth factors or the vehicle was placed in the bottom chamber, and cells in suspension (1.5×10^5^ per well in serum-free endothelial basal medium were added to the top chamber and incubated at 37°C for 4 hours. Cells that had not migrated or had only adhered to the upper side of the membrane were removed before the membrane was fixed and stained with a Reastain Quik-Diff kit (IBG Immucor Ltd, West Sussex, United Kingdom) using the manufacturer’s protocol and mounted on a glass slide. Cells that had migrated to the lower side of the membrane were counted at ×8 magnification.

### Aortic Ring Assay of Angiogenic Sprouting

The murine aortic ring angiogenesis assay was set up as described previously.^9^ In some experiments, aortic rings were infected with adenoviral constructs overnight in Opti-MEM (Life Technologies) on the day of harvest. Medium containing either no addition (control) or supplemented with 30 ng/mL VEGF, was replaced and fresh growth factors supplemented every 2 to 3 days. After 1 week, the aortic rings were fixed with 4% formalin for 30 minutes at room temperature. The fixed rings were permeabilized with PBS containing 0.25% Triton X-100 for 15 minutes (2×), blocked with casein solution for 30 minutes and incubated overnight with DyLight 594 Labeled GSL I-isolectin B4 (Vector Laboratories, 1:100) and anti-NRP1 antibody (Abcam, clone EPR3113) at 4°C. Negative controls were either incubated with PBS (for the isolectin B4-DyLight 594 antibody conjugate control) or rabbit IgG (for the Nrp1 antibody control) instead of primary antibody. Next morning, rings were washed 3× with PBS, and incubated for 1 hour in the dark with Alexa Fluor 488-conjugated donkey anti-rabbit IgG (Life Technologies), mounted on slides and imaged under a fluorescent microscope (Zeiss Axio Imager A1). The isolectin stained images were analyzed using Image J to evaluate the total area of outgrowth and number of branch points by an observer blinded to the treatments given.

### Immunofluorescent Staining

HUVECs were plated onto glass coverslips precoated with attachment factor (Life Technologies). The following day cells were infected with the adenoviral constructs and after an overnight incubation, the medium was replaced with fresh endothelial basal medium containing 10% fetal bovine serum. Forty-eight hours after infection, cells were rinsed 3× with PBS and fixed in 4% paraformaldehyde for 10 minutes followed by permeabilization with 0.1% Triton X-100 in PBS for 10 minutes. Cells were blocked in 1% BSA for 30 minutes at room temperature and incubated overnight at 4°C with C-19 antibody in PBS containing 1% BSA, or normal goat IgG for the negative control. After washing with PBS, cells were then incubated for 1 hour in the dark with Alexa Fluor 488-conjugated donkey anti-goat IgG (Life Technologies). Cells were then rinsed 3× with PBS then mounted using ProLong Gold antifade reagent with DAPI (Life Technologies). Images were acquired using a Leica TCS SP2 confocal microscope using a Planapo 63×/1.25 oil immersion objective and images were acquired in the horizontal (x-y) and in the vertical (x-z) planes by the LAS-AF software. Offline analysis was performed using ImageJ.

### VEGFR2 Phosphorylation

VEGFR2 phosphorylation was determined as described previously.^7^

### Statistical Analysis

Values have been presented as scatterplots with individual data points. Data were tested for normality using the Shapiro-Wilk test and equality of variance using the Levene test. Where necessary data were log transformed before being analyzed using either 1-way or 2-way ANOVA as appropriate with the Bonferroni correction for multiple pairwise comparisons.

## Results

### Expression of NRP1 Fragments Containing the Cytoplasmic Domain

**Figure 1.**
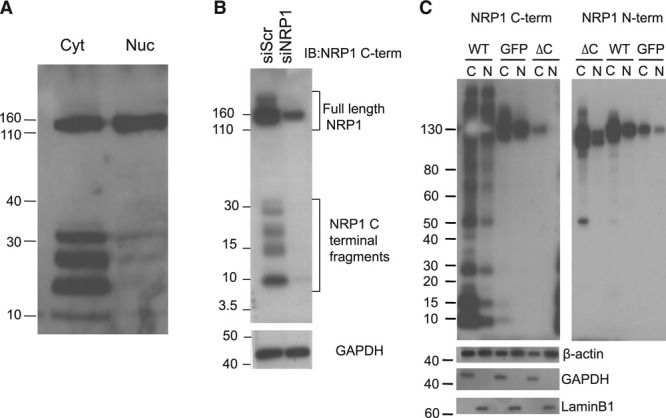
Endothelial expression of C-terminal NRP1 (neuropilin-1) fragments. **A**, Lysates of cytoplasmic (Cyt, C) and nuclear extracts (Nuc, N) of human umbilical vein endothelial cells (HUVECs) were immunoblotted with NRP1 antibody specific to the cytoplasmic domain of NRP1 (C-term; antibody C-19 from Santa Cruz Inc). This antibody recognized several low (30 kDa and below) molecular weight species predominantly in the cytoplasmic fraction. **B**, HUVECs were transfected with control scrambled (siScr) and NRP1-specific siRNAs, and whole cell protein lysates immunoblotted with NRP1 antibody specific either to the cytoplasmic (C-term) or extracellular (N-term; antibody AF387) domains of NRP1. Knockdown of endogenous NRP1 using siRNA resulted in diminished expression of not only the full-length NRP1 band but also of the cytoplasmic fragments. **C**, HUVECs were transduced with adenoviruses encoding wild-type (WT) NRP1 (WT), an NRP1ΔC mutant lacking the cytoplasmic domain (ΔC), or GFP (green fluorescent protein), and 48 h later cytoplasmic (C) or nuclear (N) cell lysates were immunoblotted with antibodies either specific for the NRP1 cytoplasmic domain (C-term), or specific to the NRP1 extracellular domain (N-term), or for β-actin, glyceraldehyde 3-phosphate dehydrogenase (GAPDH; cytoplasmic marker), or lamin B (nuclear marker). Overexpression of WT NRP1 by an adenovirus (WT), but not of the NRP1ΔC mutant (lacking the cytoplasmic domain; ΔC) results in the generation of low molecular weight cytoplasmic fragments that can be detected only by antibody specific for the NRP1 cytoplasmic domain, but not by antibody specific for the NRP1 extracellular domain.

Western blots of lysates of HUVECs with an antibody specific for the cytoplasmic domain of NRP1 detected the major full-length NRP1 protein of 130 kDa, but additionally recognized several smaller species with molecular weights of ≈10, 15, 25, and 30 kDa, primarily in the cytoplasmic compartment with less expression detected in nuclear extracts (Figure 1A). An antibody directed against the extracellular NRP1 region did not recognize these low molecular weight NRP1 species (results not shown). Immunoblots of human coronary artery smooth muscle cells and of breast cancer MB231 cells also detected several small NRP1 species recognized by antibodies specific for the cytoplasmic domain in addition to the 130 kDa full-length protein (Figure I in the online-only Data Supplement and results not shown). To examine the specificity of the NRP1 immunoreactivity of these species, NRP1 knockdown was performed using NRP1-targeted siRNA. As shown in Figure 1B, NRP1-specific siRNA depleted both the 130 kDa NRP1 protein and the low molecular weight C-terminal domain species. Furthermore, transduction of HUVECs with an adenoviral construct encoding full-length wild-type NRP1 resulted in increased expression of both a major 130 kDa band corresponding to full-length NRP1, and of 2 lower molecular weight bands, of 10, and 15 kDa, very similar in size to endogenous bands detected in HUVECs that were recognized by cytoplasmic domain antibody, but not by antibody directed to the extracellular NRP1 domain (Figure 1C). In contrast, adenoviral expression of a NRP1 mutant lacking the cytoplasmic domain (Ad.NRP1ΔC), gave rise to increased expression of a major band of ≈120 kDa that was recognized by antibodies to the extracellular NRP1 domains and corresponds to the predicted molecular weight of the NRP1ΔC protein, but did not result in expression of smaller species that could be detected by antibody specific for the cytoplasmic domain (Figure 1C). A band of ≈30 kDa was also detected by antibody specific for the NRP1 cytoplasmic domain in cells overexpressing wild-type NRP1 but not in cells overexpressing NRP1ΔC (Figure 1C).

### Cytoplasmic Domain NRP1 Fragments Are Generated via Proteolytic Cleavage by ADAMs 9 and 10

To test the possibility that expression of small C-terminal NRP1 fragments could result from proteasomal or lysosomal degradative pathways, we examined whether inhibitors of endocytotic trafficking and lysosomal or proteasomal degradation had any effect on expression of NRP1 cytoplasmic domain species.^17^ Treatment with the proteasomal inhibitor, lactacystin, caused no decrease in the level of NRP1 C-terminal fragments, but instead resulted in a marked increase in expression of the 10 and 15 kDa C-terminal NRP1 bands, which was concentration-dependent, a detectable increase in C-terminal domain species being observed at 1 µmol/L, and a greater increase at 3 and 10 µmol/L lactacystin (Figure IIA in the online-only Data Supplement). Treatment of HUVECs with chloroquine, which blocks endosomal acidification and membrane trafficking of VEGFR2 in endothelial cells,^17^ also resulted in a marked increase in the level of C-terminal NRP1 species (Figure IIB in the online-only Data Supplement). These findings indicate that NRP1 C-terminal domain species undergo endocytosis and degradation via both proteasomal and lysosomal pathways, but are not themselves the products of these degradative pathways.

**Figure 2.**
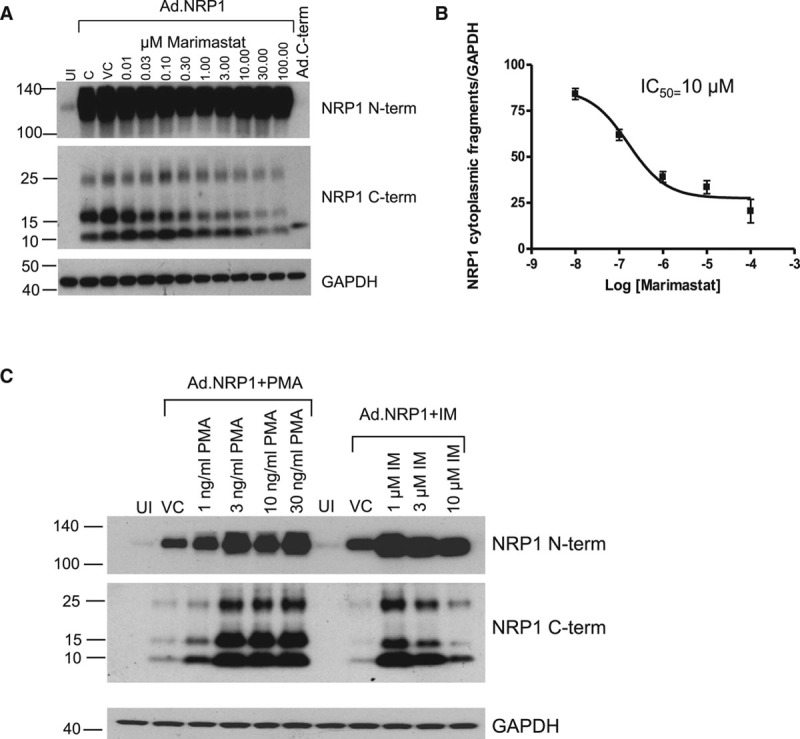
The generation of NRP1 (neuropilin-1) cytoplasmic fragments in human umbilical vein endothelial cells (HUVECs) is mediated by metalloproteinase activity. **A**, Increasing concentrations of marimastat, a broad-spectrum metalloproteinase inhibitor, reduces the expression of the 10 and 15 kDa fragments detected by antibody specific for the NRP1 cytoplasmic domain (C-term) in a dose-dependent manner. **B**, The dose-response curve for the effect of marimastat on generation of the 10 kDa NRP1 cytoplasmic fragment indicates an IC_50_ of 10 μmol\L, similar to reported values for marimastat. **C**, HUVECs, either infected with Ad.NRP1WT or uninfected (UI) were treated for 24 h with Phorbol ester (PMA), or Ionomycin (IM), or vehicle (DMSO) control (VC), at the indicated concentrations, and cell lysates were then prepared and immunoblotted as shown.

It was next investigated whether expression of C-terminal NRP1 fragments could result from posttranslational proteolytic cleavage mediated by a membrane-bound or extracellular proteinase, such as a matrix metalloproteinase or γ-secretase. Treatment with the γ-secretase inhibitor, DAPT, had no significant effect on expression of C-terminal NRP1 species, but markedly inhibited constitutive expression of the Notch cytoplasmic domain, which is produced specifically via γ-secretase cleavage (Figure IIC in the online-only Data Supplement). In contrast, treatment of HUVECs with a broad-specificity inhibitor of metalloproteinases, marimastat, significantly reduced expression of the 10 and 15 kDa NRP1 fragments (Figure 2A). This effect was concentration-dependent with a half-maximum effect (IC50) of ≈10 μmol/L marimastat (Figure 2B), similar to the reported concentration-dependent effects of this compound in intact cells.^15^ Metalloproteinase-mediated ectodomain shedding of membrane-associated molecules is stimulated by phorbol esters and calcium ionophores, which respectively activate PKCs (protein kinase C) and increase intracellular Ca^2+^.^18,19^ Because generation of NRP1 C-terminal domain species might be a consequence of NRP1 extracellular domain shedding, we therefore examined the effects of PMA and the ionophore ionomycin on expression of these protein species. As shown in Figure 2C, PMA caused a concentration-dependent increase in the level of the NRP1 10 and 15 kDa C-terminal fragments. Ionomycin also strongly increased expression of the 10 and 15 kDa NRP1 C-terminal fragments at 1 μmol/L, an effect which diminished at higher concentrations of ionomycin (Figure 2C), because of a large increase in cell detachment and loss of endothelial cell viability observed on incubation for 24 hours at concentrations >1 μmol/L.

**Figure 3.**
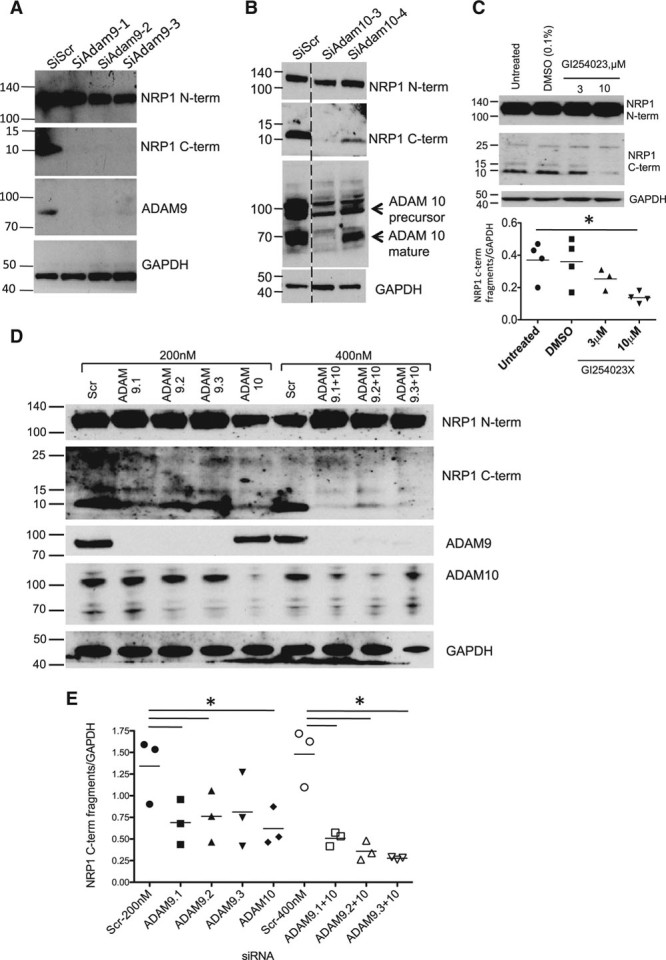
NRP1 (neuropilin-1) cleavage in endothelial cells is mediated by ADAMs (a disintegrin and metalloproteinases) 9 and 10. Human umbilical vein endothelial cells (HUVECs) were transfected with control siRNA (siScr) or siRNAs specific for ADAM9 (**A**) or ADAM10 (**B**), and after 72 h were then treated with 25 ng/mL VEGF (vascular endothelial growth factor) for 60 min; cell lysates were then immunoblotted with the antibodies indicated. **C**, HUVECs were treated for 24 h with either no additions, or with DMSO (vehicle), or with the indicated concentrations of GI254023X, a specific inhibitor of ADAM10, and cell lysates were then immunoblotted with the antibodies indicated; quantification of the 10 kDa NRP1 cytoplasmic domain band is shown, *P<0.05 vs DMSO treatment, n≥3. **D**, Effects of single and double knockdown of ADAM9 and ADAM10 on the expression of NRP1 cytoplasmic domain fragments. The blots shown are representative of 4 different experiments. **E**, Quantification of the 10 kDa NRP1 cytoplasmic fragment from experiments in **D**, normalized to glyceraldehyde 3-phosphate dehydrogenase (GAPDH) expression, after knockdown of ADAMs 9 or 10 using single siRNAs at 200 nmol/L (black symbols), or double knockdown of ADAM9 plus ADAM10 (unfilled symbols) using combinations of siRNAs (total siRNA concentration 400 nmol/L); *P<0.05 vs Scr siRNA (200 nm) or Scr siRNA (400 nm) as appropriate. Values are presented as a scatterplot. Differences between samples were analyzed using 1-way ANOVA with the Bonferroni correction for multiple pairwise comparisons after testing for normality and equal variance using the Shapiro-Wilk and Levene tests, respectively.

These findings suggested that NRP1 cytoplasmic domain fragments could be generated from full-length NRP1 by proteolytic cleavage mediated via an extracellular or membrane-associated protease, such as a member of the ADAM family. ADAMs family members that are expressed and shown to have functional roles in endothelial cells include ADAMs 9, 10, 15, and 17,^16,20,21^ and we therefore focused on these ADAMs. Knockdown of ADAM9 or ADAM10 using specific siRNAs caused a marked and significant reduction in the endogenous level of NRP1 C-terminal fragments (Figure 3A and 3B). In contrast, targeted depletion of ADAM17 had little effect on expression of either full-length NRP1 or NRP1 C-terminal species (Figure III in the online-only Data Supplement). Knockdown of ADAM15 also caused no significant reduction in expression of NRP1 C-terminal species (data not shown). To further validate the conclusion that NRP1 C-terminal domain fragments were generated via an ADAM-dependent pathway, we also examined the effect of the specific ADAM10 inhibitor, GI254023X.^22^ Treatment of endothelial cells with GI254023X markedly reduced endogenous generation of 10 and 15 kDa NRP1 C-terminal bands (Figure 3C). The effect of GI254023X was concentration-dependent with a decrease in expression of the 10 and 15 kDa NRP1 C-terminal bands detectable at 3 μmol/L and a more striking reduction observed at 10 μmol/L. We also examined the effects of double knockdown of ADAMs 9 and 10. As indicated in Figures 3D and 3E, targeted depletion of both ADAMs caused a more marked decrease in the level of 10 and 15 kDa NRP1 C-terminal bands compared with the effects of single knockdowns.

### VEGF Regulation of Cytoplasmic domain NRP1 fragments via ADAMs 9 and 10

**Figure 4.**
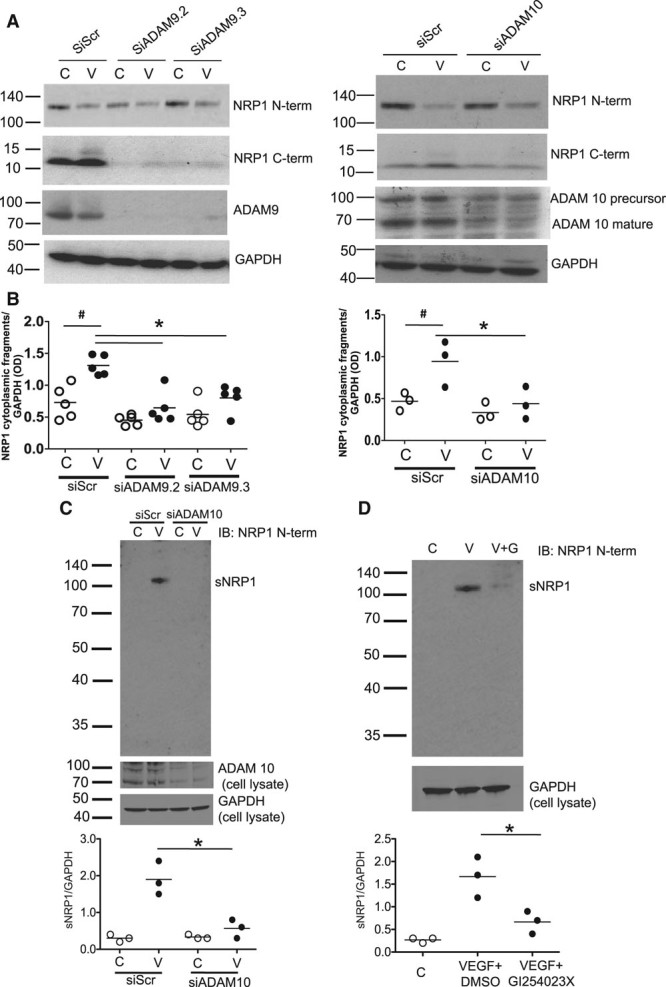
ADAMs (a disintegrin and metalloproteinases) 9 and 10 mediate VEGF (vascular endothelial growth factor)-induced NRP1 (neuropilin-1) proteolytic cleavage. **A**, Human umbilical vein endothelial cells (HUVECs) were transfected with siRNAs as indicated, and after 72 h were then treated with 25 ng/mL VEGF-A for 60 min, and lysates were then immunoblotted as indicated. **B**, Quantification of the 10 kDa NRP1 cytoplasmic domain fragment in experiments in **A** in which HUVECs were treated without (C, unfilled symbols) or with VEGF-A (V, black symbols); VEGF significantly enhanced the generation of NRP1 cytoplasmic fragments in siScr-treated cells and this was blocked by siADAM9 (**left** graph) or siADAM10 (**right** graph); #*P*<0,05 vs siScr C, *P<0.05; **P<0.01; ***P<0.005 vs siScr plus VEGF (V; n≥3). **C**, HUVECs were either transfected with control (siScr) and ADAM10 specific siRNAs and were then either untreated (C, control, unfilled symbols)) or treated with 25 ng/mL VEGF (V, black symbols) for 60 min. Cell supernatant was then removed, concentrated, and immunoblotted NRP1 antibody specific for the extracellular domain with (AF3870) to detect sNRP1 (soluble NRP1). Quantification of results from 3 independent experiments is shown below; **P<0.01 for siADAM10 vs siScr. **D**, HUVECs were untreated (C, control, unfilled symbols), or were treated for 60 min with 25 ng/mL VEGF plus either DMSO (V, black symbols) or with 25 ng/mL VEGF plus an equal volume of GI254023X (V+G, black symbols). Quantification of results from 3 independent experiments is shown below; *P<0.05 for VEGF plus GI254023X (10 μmol/L) vs VEGF plus DMSO. In this figure, values are presented as a scatterplot. Differences between samples were analyzed using 1-way ANOVA with the Bonferroni correction for multiple pairwise comparisons after testing for normality and equal variance using the Shapiro-Wilk and Levene tests, respectively.

Our findings supported the conclusion that generation of NRP1 C-terminal domain fragments was the consequence of a constitutive proteolytic process mediated via ADAMs 9 and 10. To investigate whether formation of these fragments was regulated by physiological stimuli, we tested the effects of VEGF on generation of NRP1 C-terminal domain species. As shown in Figure 4A, VEGF increased formation of the 10 kDa NRP1 C-term species, with a consistent and significant increase in expression of 10 and 15 kDa NRP1 C-terminal bands after 1-hour treatment with VEGF. Furthermore, knockdown of either ADAM9 or ADAM10 blocked the VEGF-induced increase in generation of these C-terminal domain fragments (Figure 4B). VEGF treatment also caused a marked and significant increase in generation of a soluble extracellular fragment of NRP1 (sNRP1) of ≈120 kDa, consistent with previous findings,^16^ which was detected in endothelial cell supernatant using antibody specific for the NRP1 ectodomain (Figure 4C; Figure IV in the online-only Data Supplement). VEGF-induced NRP1 ectodomain shedding was also inhibited by knockdown of ADAM10 and by ADAM10 inhibition using GI254023X, indicating that ADAM10 cleavage of NRP1 resulted in generation of a sNRP1 containing most of the extracellular domain comprising the VEGF and Sema3 ligand-binding regions, and NRP1 species containing the cytoplasmic, and possibly other domains including the transmembrane and MAM domains. We also examined the possibility that generation of NRP1 C-terminal domain fragments could be regulated by other cytokines. Our studies showed that treatment of endothelial cells with TNF (tumor necrosis factor)-α for 24 hours also significantly increased expression of the major 10 kDa NRP1 C-terminal fragment (Figure V in the online-only Data Supplement).

**Figure 5.**
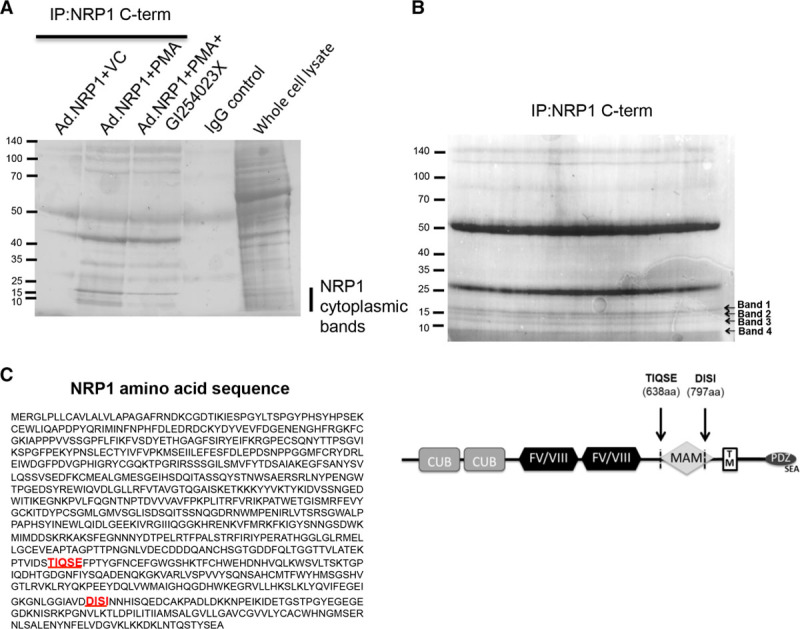
Protein sequencing of NRP1 (neuropilin-1) cleavage products. **A**, Human umbilical vein endothelial cells (HUVECs) were transduced with Ad.NRP1WT and after 48 h were treated with 30 ng/mL PMA for 24 h in the absence or presence of the ADAM (a disintegrin and metalloproteinase) 10 inhibitor, GI254023X (10 μmol/L). Cells were lysed, immunoprecipitated using antibody specific for the NRP1 cytoplasmic domain (IP: NRP1 C-term; C-19), and proteins were stained with Ponceau S to detect bands. In parallel, HUVECs transduced with Ad.NRP1WT for 48 h were lysed and lysates incubated with IgG control. Whole cell lysate of PMA-treated Ad.NRP1WT-infected cells is also shown for comparison. **B**, HUVECs were transduced with Ad.NRP1WT and after 48 h were treated with 30 ng/mL PMA for 24 h. Cells were then lysed and full-length NRP1 and the low molecular weight cleavage products were immunoprecipitated using the C-19 antibody specific for the NRP1 cytoplasmic domain, and immunoprecipitated proteins separated by SDS-PAGE. Proteins were transferred onto a PVDF membrane and stained with Ponceau S to detect bands. The low molecular weight cleavage products, indicated as bands 1 to 4, were cut and sent for N-terminal sequencing by Edman degradation. **C**, Amino acid sequence of NRP1, N-terminal sequences detected by N-terminal sequencing by Edman degradation are highlighted in red within the NRP1 sequence. A schematic diagram representing the putative sites of NRP1 cleavage by members of the ADAM family, resulting in the generation of cytoplasmic fragments is shown.

Our data indicated that expression of low molecular weight NRP1 C-terminal species was the result of ADAM-mediated cleavage of full-length membrane-associated NRP1 giving rise to 2 major NRP1 C-terminal species of ≈10 and 15 kDa. Based on molecular weight and immunoreactivity, these species were predicted to contain the cytoplasmic and transmembrane domains plus part of the juxtamembrane and MAM extracellular regions. To obtain further insight into the cleavage sites utilized by ADAM 9 and 10 giving rise to these species, we sought to obtain protein sequence data for these C-terminal species in endothelial cells. To obtain sufficient protein for sequencing, the expression of NRP1 C-terminal cleavage products was increased by adenovirally overexpressing NRP1 in endothelial cells and further stimulating cleavage by treatment with PMA (Figure 2C). NRP1 was then immunoprecipitated using antibody specific to the C-terminal domain, and potential cleavage products were identified by Ponceau S staining. It was confirmed that expression of 10 and 15 kDa NRP1 fragments immunoprecipitated by antibody specific to the NRP1 C-terminal domain and detected by Ponceau S staining was inhibited by treatment of cells with the ADAM10-specific inhibitor, GI254023X (Figure 5A). Edman degradation sequencing of Ponceau S stained NRP1 fragments immunoprecipitated by antibody specific to the NRP1 C-terminal domain (Figure 5B) showed that the 10 and 15 kDa NRP1 bands had N-terminal sequences comprising, respectively, **DISI** (Asp Iso Ser Iso) juxtamembrane to the MAM domain, and **TIQSE** (Thr Iso Gln Ser Glu) near the N-terminal end of the MAM domain (Figure 5C). These findings suggest that ADAMs 9 and 10 cleave NRP1 in its extracellular region resulting in at least 2 species comprising either the cytoplasmic, transmembrane, and juxtamembrane domains, or the cytoplasmic, transmembrane, juxtamembrane, and MAM domains (Figure 5C, right).

### C-Terminal Domain NRP1 Fragments Inhibit VEGF Angiogenic Signaling in Endothelial Cells

**Figure 6.**
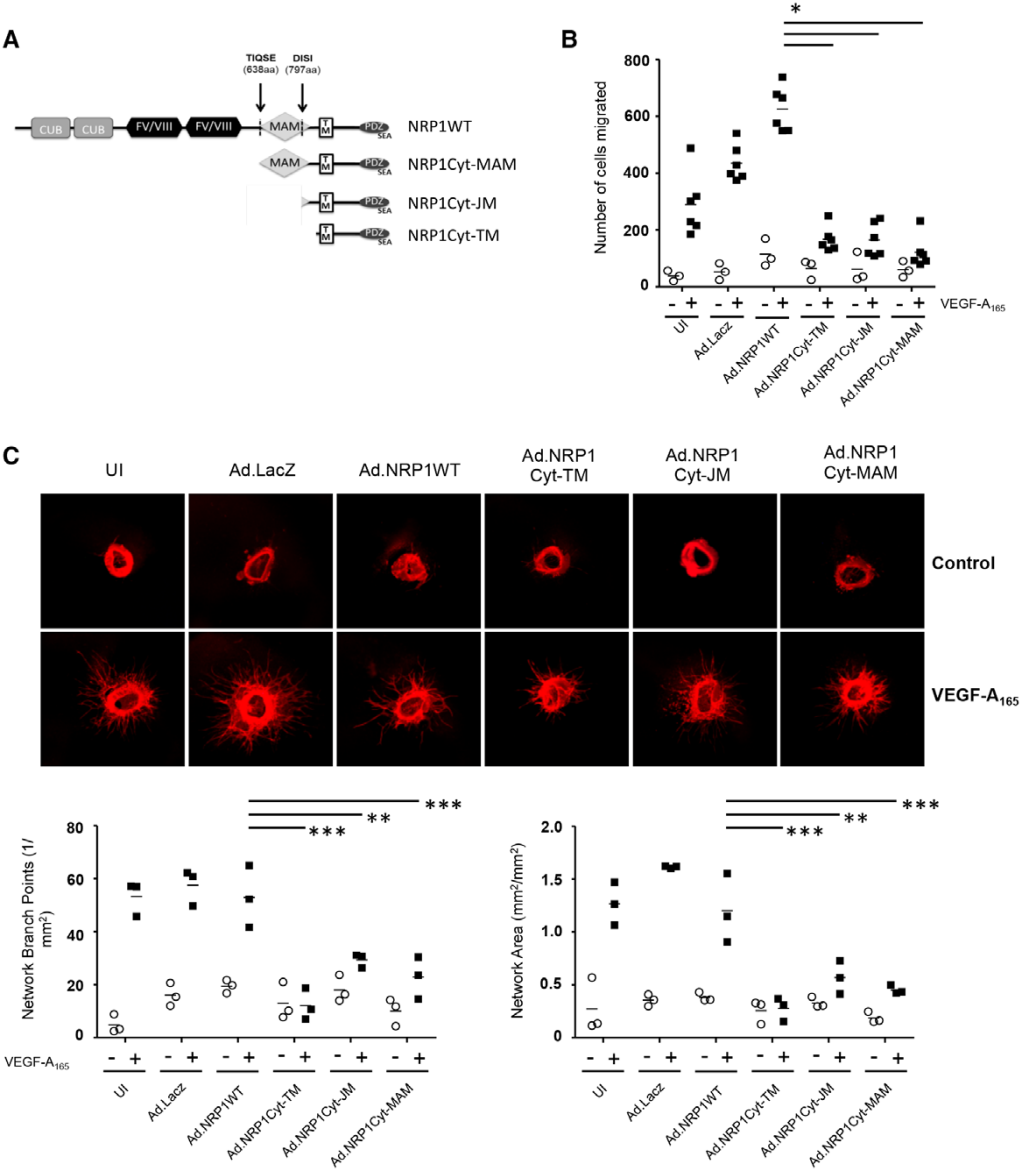
Overexpression of NRP1 (neuropilin-1) cytoplasmic domain fragments in human umbilical vein endothelial cells (HUVECs) inhibits VEGF (vascular endothelial growth factor)-induced angiogenesis. **A**, Schematic diagram representing adenoviral NRP1 constructs comprising the cytoplasmic and transmembrane domains (Ad.NRP1Cyt-TM, residues 860–923), the cytoplasmic, transmembrane, and juxtamembrane regions (Ad.NRP1Cyt-JM, residues 797–923), and the cytoplasmic, transmembrane, juxtamembrane, and MAM domains (Ad.NRP1Cyt-MAM, residues 638–923). **B**, HUVECs transfected with adenoviruses overexpressing LacZ, wild-type NRP1 (Ad.NRP1WT), or the cytoplasmic domain species (Ad.NRP1Cyt-TM, Cyt-JM and Cyt-MAM, respectively) were used in a Transwell migration assay with (+, black symbols) and without (−, unfilled symbols) VEGF treatment (25 ng/mL, 4 h); the means±SEM of results from 3 independent assays are shown, *P*<0.05 vs Ad.NRP1WT plus VEGF. **C**, Aortic rings were incubated with the indicated adenoviruses in Opti-MEM overnight. The aortic ring assay was performed as detailed in the Materials and Methods with no treatment (−, unfilled symbols) or with VEGF-A_165_ treatment (+, black symbols). Quantification of the number of branch points (**left** graph) and network area (**right** graph) are shown below the representative figures; **P<0.01, ***P<0.001 vs Ad.NRP1 plus VEGF, n=3 (each n includes aortic rings from 4 mice, to have sufficient sample to set up each condition using 6 replicate aortic rings). In this figure, values are presented as a scatterplot. Differences between samples were analyzed using 2-way ANOVA with the Bonferroni correction for multiple pairwise comparisons after testing for normality and equal variance using the Shapiro-Wilk and Levene tests, respectively.

To investigate whether NRP1 C-terminal domain species generated by ADAM-dependent cleavage could exert functional effects in HUVECs independently of the NRP ligand-binding domain, we generated adenoviral (Ad) constructs encoding either NRP1WT (Ad.NRP1), or different NRP1 deletion mutants, and compared their effects on VEGF angiogenic signaling in endothelial cells. The constructs tested comprised either the cytoplasmic and transmembrane domains (Ad.NRP1Cyt-TM, residues 860–923), the cytoplasmic, transmembrane, and juxtamembrane regions (Ad.NRP1Cyt-JM, residues 797–923), or the cytoplasmic, transmembrane, juxtamembrane, and MAM domains (Ad.NRP1Cyt-MAM, residues 638–923; Figure 6A). Expression of all constructs in HUVECs was confirmed by Western blot (data not shown) and by immunofluorescent staining (Figure VI in the online-only Data Supplement). NRP1 is strongly implicated in mediating VEGF stimulation of endothelial cell migration.^3–5,7,8^Therefore, initially we determined effects of NRP1 cytoplasmic domain-containing constructs in assays of VEGF-stimulated endothelial cell chemotactic migration. As shown in Figure 6B, in endothelial cells expressing Ad.NRP1WT or Ad.LacZ, VEGF induced a striking chemotactic response, whereas cells expressing either Ad.NRP1Cyt-TM, Ad.NRP1Cyt-JM, or Ad.NRP1Cyt-MAM exhibited a significantly reduced migratory response to VEGF compared with cells expressing either Ad.NRP1WT or Ad.LacZ. In contrast, none of the adenoviruses expressing either WT NRP1 or truncated C-terminal NRP1 fragments had any effect on endothelial cell proliferation either with or without addition of VEGF, as determined by real-time measurement of cell confluence >72 hours (data not shown). This is in agreement with previous findings showing that NRP1 is not required for endothelial cell proliferation.^23^ The effects of overexpressing NRP1 C-terminal domain species were next examined in an aortic ring assay of sprouting angiogenesis. VEGF induced new angiogenic sprouts in aortic rings transduced with either Ad.NRP1WT or Ad.LacZ, as determined by endothelial-specific staining and measurement of both branch points and total endothelial network area (Figure 6C). NRP1 expression in the endothelial cells of newly sprouted vessels was confirmed in aortic rings by co-immunofluorescent staining with the endothelial cell-specific marker isolectin B4 (Figure VII in the online-only Data Supplement). In contrast, VEGF-induced angiogenesis was markedly reduced in aortic rings expressing either Ad.NRP1CytTM, Ad.NRP1CytJM, or Ad.NRP1CytMAM as compared with control aortic rings expressing Ad.NRP1WT or Ad.LacZ. We further examined the effect of NRP1 C-terminal domain constructs on angiogenesis in the coculture model of angiogenesis. Similar to results obtained in the aortic ring assay, overexpression of either Ad.NRP1CytTM, Ad.NRP1CytJM, or Ad.NRP1CytMAM, reduced endothelial network formation in the coculture angiogenesis assay in comparison to Ad.NRP1WT overexpression (data not shown).

**Figure 7.**
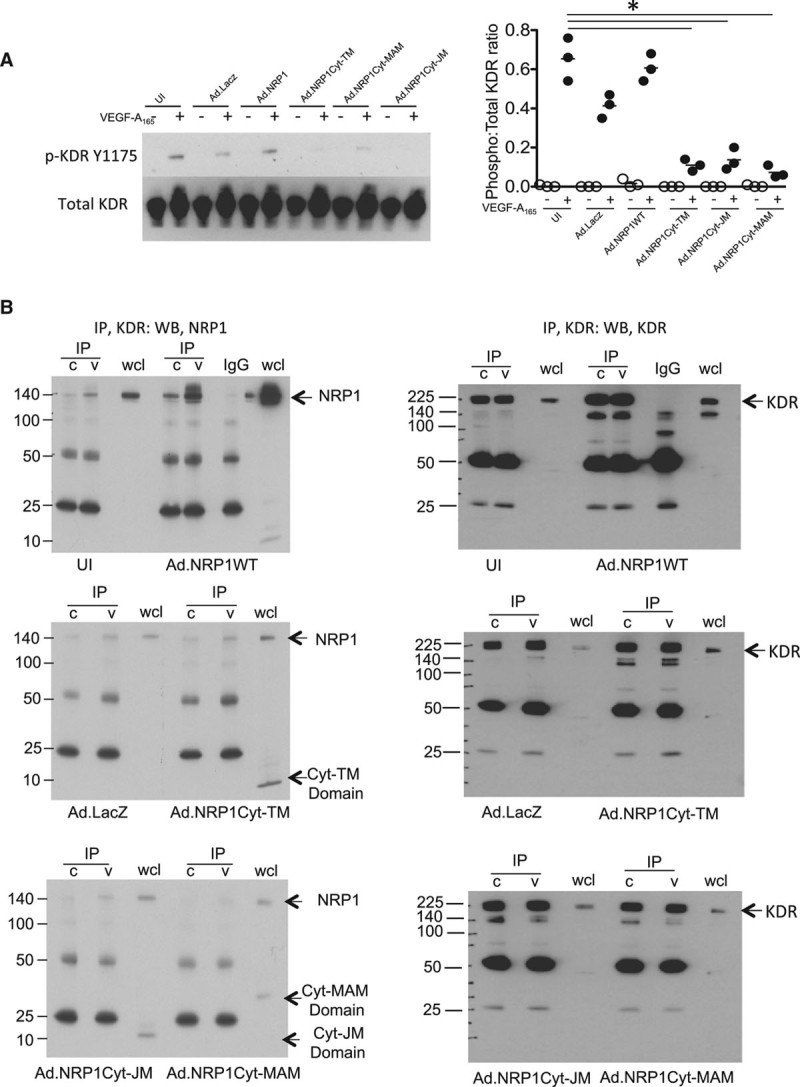
Overexpression of NRP1 (neuropilin-1) cytoplasmic fragments in human umbilical vein endothelial cells (HUVECs) inhibits KDR (kinase domain insert receptor) activation but not VEGF (vascular endothelial growth factor)-induced NRP1 association with KDR. **A**, HUVECs were transfected with adenoviruses overexpressing LacZ, wild-type NRP1 (Ad.NRP1WT), or the low molecular weight species (Ad.NRP1Cyt-TM, Cyt-JM, and Cyt-MAM, respectively). After treatment without (−, unfilled symbols) or with VEGF-A_165_ (+, black symbols) for 10 min, cells were lysed and immunoblotted with the indicated antibodies. Quantification of the blots is shown on the right for KDR (n=3; *P<0.05 vs Ad.NRP1WT plus VEGF). **B**, HUVECs overexpressing WT and mutant NRP1s as detailed above were stimulated with VEGF for 10 min and KDR was immunoprecipitated. Immunoprecipitates were immunoblotted with an antibody to NRP1. Blots demonstrating equal KDR immunoprecipitation for each condition are shown on the right-hand side. In this figure, values are presented as a scatterplot. Differences between samples were analyzed using 2-way ANOVA with the Bonferroni correction for multiple pairwise comparisons after testing for normality and equal variance using the Shapiro-Wilk and Levene tests, respectively.

The major angiogenic signaling receptor for VEGF in endothelial cells is VEGFR2/KDR. We next examined whether the inhibitory effects of NRP1 cytoplasmic domain constructs on VEGF-induced angiogenesis could be explained in part by reduced VEGFR2/KDR activation in response to VEGF. As shown in Figure 7A, Ad.NRP1Cyt-TM, Ad.NRP1Cyt-JM, or Ad.NRP1Cyt-MAM constructs inhibited VEGF-induced VEGFR2/KDR activation, compared with the effect of VEGF in cells expressing Ad.NRP1WT or Ad.LacZ. VEGF binding to NRP1 and VEGFR2 induces formation of a complex between NRP1 and VEGFR2, which is considered to play an important role in NRP1-dependent VEGF signaling in the endothelium. We next examined the possibility that NRP1 cytoplasmic domain species were able to associate with VEGFR2 independently of the extracellular ligand-binding domain, by comparing VEGFR2 association with NRP1 in cells expressing Ad.NRP1WT, Ad.NRP1Cyt-TM, Ad.NRP1Cyt-JM, or Ad.NRP1Cyt-MAM. VEGF treatment of HUVECs overexpressing NRP1WT stimulated association of VEGFR2/KDR with NRP1 as demonstrated by detection of a 130 kDa NRP1 band by Western blotting for NRP1 in VEGFR2 immunoprecipitates (Figure 7B). In cells overexpressing either NRP1Cyt-TM, NRP1Cyt-JM, or NRP1Cyt-MAM, Western blot of VEGFR2 immunoprecipitates with an NRP1 antibody specific for the C-terminal domain detected a 10 kDa band corresponding to NRP1Cyt-TM, but at a much lower level relative to full-length NRP1 (compare Figure 7B with Figure VII in the online-only Data Supplement). Furthermore, VEGF treatment caused no change in the degree of VEGFR2 co-immunopreciptation with small C-terminal domain NRP1 fragments (Figure VIII in the online-only Data Supplement). These findings indicate that the NRP1 C-terminal domain alone, independent of the NRP1 ligand-binding domain associates with VEGFR2 only weakly and that any association is not regulated by VEGF.

## Discussion

Here, we report the identification of novel NRP1 protein species containing the cytoplasmic domain and the transmembrane domain, but lacking the regions of the extracellular domain essential for binding of VEGF and semaphorin ligands. We show that these species are endogenously expressed in human endothelial, vascular smooth muscle, and tumor cells. Importantly, we demonstrate that expression of these species occurs via a VEGF-regulated and ADAMs-mediated pathway resulting in NRP1 ectodomain cleavage and generating novel NRP1 species containing the C-terminal domain. We further identify proteolytic cleavage of NRP1 by ADAM 9 and 10 as the major mechanism mediating generation of these NRP1 fragments. These conclusions are supported by the following evidence: (1) siRNAs targeted to either ADAMs 9 and 10 significantly reduced endogenous and VEGF-induced expression of NRP1 C-terminal 10 and 15 kDa domain species, whereas ADAM17 knockdown had no effect; (2) generation of NRP1 C-terminal 10 and 15 kDa domain species was inhibited by the broad-specificity metalloproteinase inhibitor, marimastat, and by the specific pharmacological ADAM10 inhibitor, GI254023X; (3) levels of NRP1 C-terminal 10 and 15 kDa domain species were increased by PMA and ionomycin, known activators of ADAM-like proteolytic activity, whereas perturbation of alternative mechanisms, including degradative pathways and γ-secretase, did not inhibit expression of these fragments; and (5) the N-terminal sequences of NRP1 C-terminal fragments generated by cleavage were similar to known consensus sites of ADAMs cleavage.

Protein sequencing of NRP1 C-terminal fragments was consistent with cleavage by ADAMs 9 and 10 at 2 sites at the juxtamembrane and N-terminal ends of the extracellular MAM domain. Furthermore, the sequences at these sites contain the motif AVD_DIS characteristic of other predicted ADAMs cleavage sites. The consensus sites of ADAMs cleavage are relatively poorly characterized,^24,25^ and there are few unambiguously identified sites of ADAM proteolysis. However, the protein sequencing of the 2 major C-terminal domain NRP1 fragments detected in endothelial cells, taken together with the fact that ADAM9/10 knockdown and ADAM10 inhibition strongly inhibited generation of these NRP1 fragments in endothelial cells indicates that NRP1 undergoes ADAM9/10-mediated cleavage at or near these sites (Figure 5). Whether ADAM9 and 10 have preference for cleavage at different sites is unclear, and knockdown of either ADAMs significantly reduced formation of the major 10 kDa C-terminal band. It is also unclear whether ADAMs 9 and 10 work processively. Unambiguous answers to these questions could not be determined from ADAM knockdowns.

Ectodomain shedding of cell surface molecules has emerged as a major pathway regulating the activity of several cell surface molecules with key roles in endothelial cell function, including VE-cadherin.^20,26,27^ Ectodomain shedding of VEGFR2 by ADAM17,^15^ or by both ADAM10 and ADAM17^22^ was previously reported. Furthermore, Donners et al^22^ also showed that ADAM10 and VEGFR2 can complex with each other, that VEGF increases ADAM10 activity, and that pharmacological inhibition of ADAM10 using GI254023X inhibited VEGF-induced endothelial cell migration. In contrast, much less is known about shedding of other endothelial VEGF receptors. Shedding of the NRP1 extracellular domain in endothelial cells has not previously been described, though Swendeman et al^15^ reported cleavage of the NRP1 ectodomain in COS-7 cells in response to ionomycin, and this study also implicated ADAM10 in this process by demonstrating enhancement of NRP1 shedding by expression of ADAM10, but not an inactive ADAM10 mutant, in ADAM10-deficient mouse embryo fibroblasts. The findings presented here, identify NRP1 as a novel substrate for ADAMs 9 and 10 in endothelial cells, and show for the first time that VEGF can regulate NRP1 by enhancing ADAM 9 and 10-mediated NRP1 cleavage leading to increased generation of NRP1 C-terminal 10 and 15 kDa domain species. Consistent with this observation, VEGF increases ADAM10 activity.^22^

NRP1 cleavage mediated via ADAM9/10 could play an important functional role in regulating the function of NRP1 in endothelial biology. During mouse and chick embryonic development, loss of axonal responsiveness to Sema3A correlates with a sharp decrease in axonal NRP1 expression.^16^ This developmental downregulation of *Nrp1* is blocked by genetic ablation of ADAM10 and ADAM17, demonstrating an important role of ADAM-mediated NRP1 cleavage in physiological regulation of axonal guidance cues. NRP1 ectodomain shedding is a potential mechanism through which NRP1-dependent VEGF signaling could be downregulated, through the decoy role of sNRP1, which might result in a dampening of the endothelial chemotactic and angiogenic responses to VEGF. Negative feedback regulation of VEGF signaling through ADAM-mediated VEGFR and NRP1 ectodomain shedding could be important for calibrating VEGF responsiveness to achieve a physiologically normal biological effect. Consistent with this notion is the finding that pharmacological inhibition of ADAM10 impairs endothelial cell migration,^22^ and that endothelial-specific ADAM10 knockout in mice results in aberrant organ-specific vascularization, including increased retinal vascular branching and density. VEGF-induced NRP1 cleavage via ADAM10 could also potentially regulate NRP1 function by causing a reduction in the total cellular level of full-length NRP1. However, because VEGF regulation of cellular NRP1 levels can also occur via ligand-induced receptor-mediated endocytosis,^28^ and may additionally be influenced by other processes such as receptor recycling and de novo synthesis, the extent of any contribution of ADAM-mediated cleavage to regulation of full-length NRP1 is unclear. Our study does not preclude involvement of other ADAMs family members in regulating NRP1, a possibility supported by our observation that TNF-α also induces NRP1 cleavage. Further work will be required to fully elucidate the ADAMs able to mediate NRP1 proteolytic cleavage in endothelial cells.

Studies of VEGF receptor processing to date have tended to focus either on vesicular trafficking, or on the role of either shed or alternatively expressed extracellular domains as potential functional regulatory mechanisms, either through negative regulation exerted via loss of functional ligand-binding domains and through the inhibitory decoy role of these soluble extracellular regions. However, ectodomain cleavage of receptors followed by intracellular juxtamembrane cleavage generates intracellular regions which have essential biological functions, generation of the Notch receptor cytoplasmic domain being one important example. Previous findings have revealed an important role for the NRP1 cytoplasmic C-terminal PDZ-domain–binding motif in regulating endothelial cell migration and angiogenesis.^29,30^ The findings presented here that NRP1 fragments containing either the cytoplasmic or the cytoplasmic, transmembrane and MAM domains but lacking the extracellular domain, significantly diminished VEGF-induced migration, sprouting angiogenesis in an ex vivo model, and VEGFR2 activation, indicate that NRP1 species unable to bind VEGF ligands can regulate angiogenic signaling. ADAMs processing of NRP1 may function as a regulatory feedback mechanism to fine-tune cellular responsiveness to ligands for NRP1 or for NRP1 coupled receptors such as VEGFR2. Previous work reported that the cytoplasmic PDZ-binding domain of NRP1 is essential for NRP1 complex formation with VEGFR2.^31^ Further work will be necessary to demonstrate whether NRP1 cytoplasmic domain fragments generated by ADAMs-mediated cleavage can regulate functional or pathological angiogenesis in an in vivo setting.

However, the weak association of NRP1 species containing the cytoplasmic domain but lacking the ligand-binding domain with VEGFR2, and the lack of VEGF stimulation of association of these species with VEGFR2, indicates that the cytoplasmic domain is insufficient for complexation with VEGFR2 in the absence of the ligand-binding region. The dominant negative effect of cytoplasmic domain species on VEGF signaling via VEGFR2 to stimulate cell migration and angiogenesis seems therefore to be exerted through an indirect mechanism. The NRP1 cytoplasmic domain associates with intracellular signaling molecules such as synectin and p130Cas, and these associations may be important for mediating the role of NRP1 in angiogenesis and cell migration.^32,33^ Thus, the inhibitory effect of NRP1 constructs containing the cytoplasmic domain and lacking the ligand-binding region may be because of binding and sequestration of the NRP1 cytoplasmic domain to intracellular signaling mediators that are essential for normal VEGF angiogenic signaling. These findings suggest that the ADAMs-regulated balance between full-length NRP1 and cell-associated fragments lacking the ligand-binding domain may be important for determining angiogenic signaling in response to VEGF.

## Sources of Funding

This study was supported by British Heart Foundation (BHF) program grant RG/06/003 (I. Zachary, I.M. Evans, V. Mehta, M. Mahmoud) and BHF Project grant PG/12/65/29840 (to I. Zachary and C. Pellet-Many), and by funding from the European Union’s Seventh Program for research, technological development, and demonstration under grant agreement no. 278313, BIOMAGSCAR (V. Mehta and L. Fields).

## Disclosures

None.

## Supplementary Material

## Nonstandard Abbreviations and Acronyms

Ad: adenoviruses
ADAM: a disintegrin and metalloproteinase
HUVEC: human umbilical vein endothelial cells
NRP1: neuropilin-1
siRNA: small interfering RNA
sNRP1: soluble NRP1
TNF: tumor necrosis factor
VEGF: vascular endothelial growth factor
VEGFR2: VEGF receptor tyrosine kinase

## Notes

Highlights

- NRP1 (neuropilin-1) undergoes cleavage in endothelial cells to generate novel fragments containing the cytoplasmic domain but lacking the ligand-binding region.
- These novel species containing the cytoplasmic domain of NRP1 are generated via proteolytic cleavage by ADAMs (a disintegrin and metalloproteinases) 9 and 10.
- VEGF (vascular endothelial growth factor) induces formation of novel NRP1 species in endothelial cells containing the cytoplasmic domain via ADAMs 9 and 10.
- Overexpression of the novel cytoplasmic fragments of NRP1 in endothelial cells negatively regulates VEGF angiogenic signaling.
