# Supplementary figures and images for: VEGF (Vascular Endothelial Growth Factor) Induces NRP1 (Neuropilin-1) Cleavage via ADAMs (a Disintegrin and Metalloproteinase) 9 and 10 to Generate Novel Carboxy-Terminal NRP1 Fragments That Regulate Angiogenic Signaling
Source: Arterioscler Thromb Vasc Biol. 2018 Jun 7;38(8):1845–58. doi: 10.1161/ATVBAHA.118.311118 (PMC6092111; doi:10.1161/ATVBAHA.118.311118)

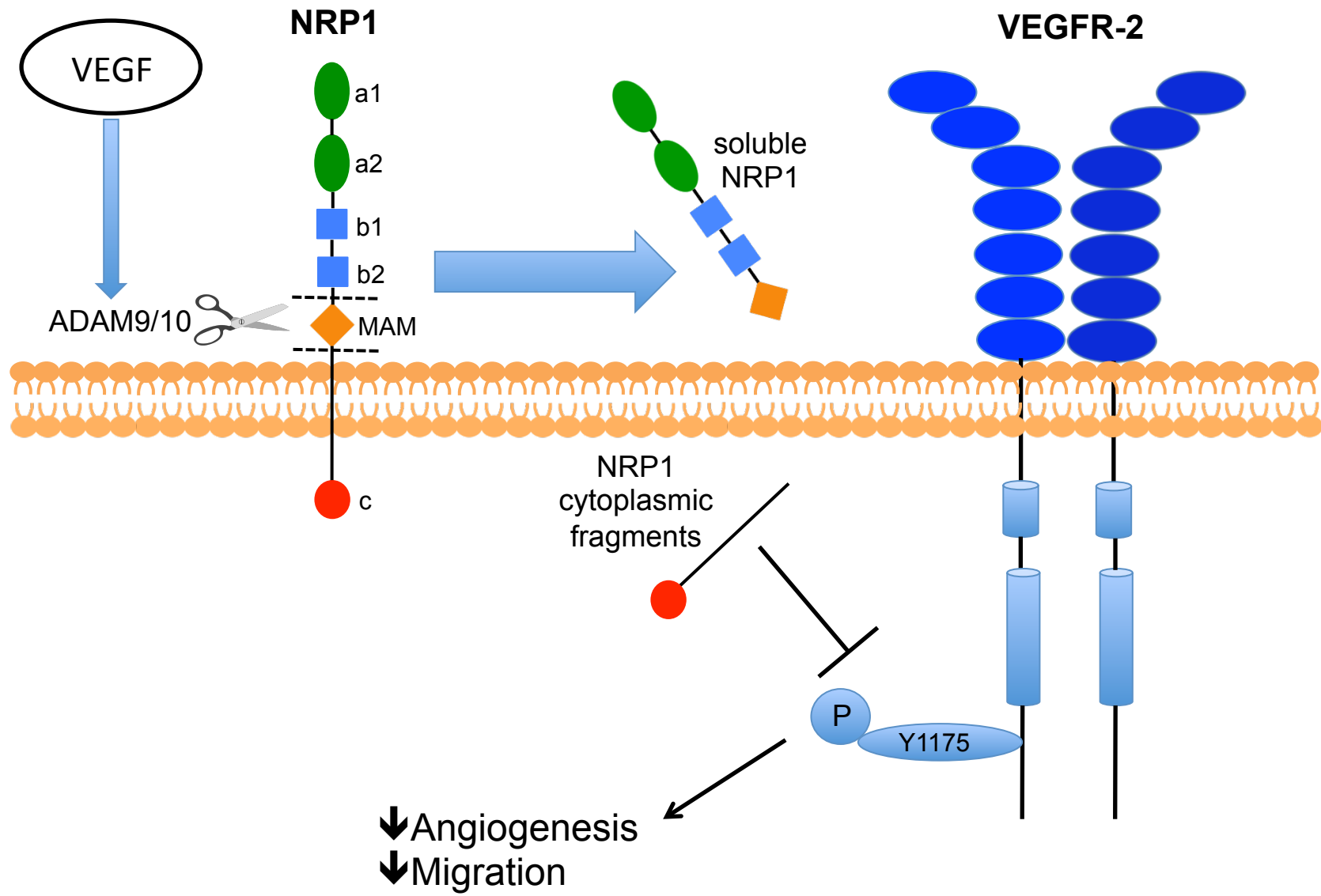

Supplement: Supplementary file 1 [file atv-38-1845-s001.pdf]
