# Supplementary material for: VEGF (Vascular Endothelial Growth Factor) Induces NRP1 (Neuropilin-1) Cleavage via ADAMs (a Disintegrin and Metalloproteinase) 9 and 10 to Generate Novel Carboxy-Terminal NRP1 Fragments That Regulate Angiogenic Signaling
Source: Arterioscler Thromb Vasc Biol. 2018 Jun 7;38(8):1845–58. doi: 10.1161/ATVBAHA.118.311118 (PMC6092111; doi:10.1161/ATVBAHA.118.311118)
Supplement: Supplementary file 2 [file atv-38-1845-s002.pdf]

Supplementary figures

Figure I

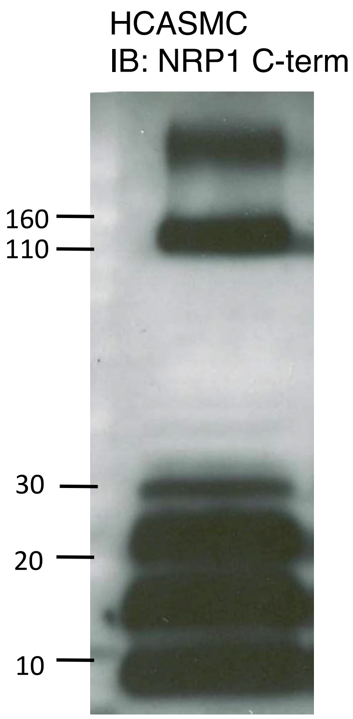

Figure II

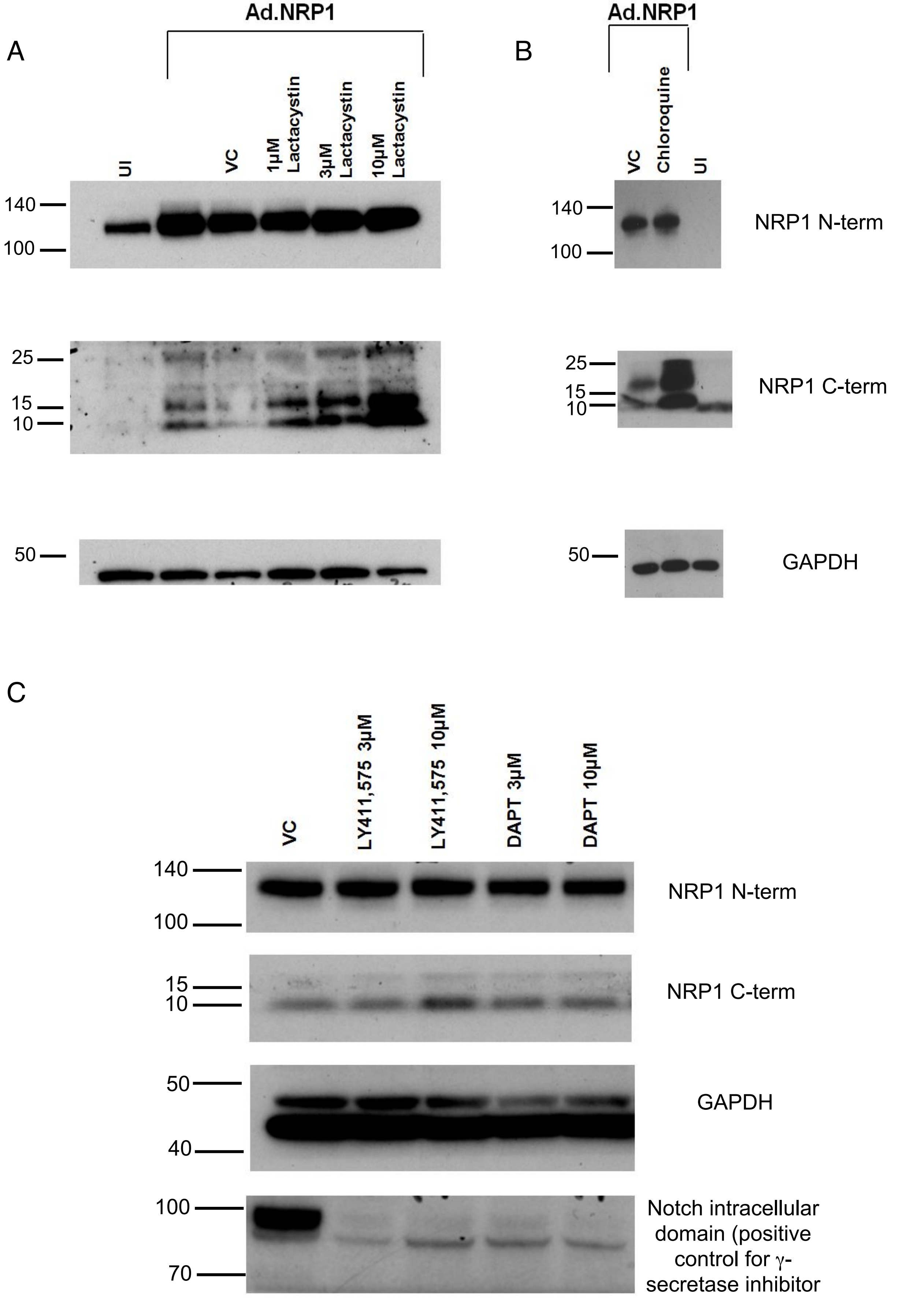

A
Ad.NRP1
UI
VC
1µM Lactacystin
3µM Lactacystin
10µM Lactacystin
140
100
25
15
10
50
B
Ad.NRP1
VC
Chloroquine
UI
140
100
NRP1 N-term
25
15
10
NRP1 C-term
50
GAPDH
C
VC
LY411,575 3µM
LY411,575 10µM
DAPT 3µM
DAPT 10µM
140
100
NRP1 N-term
15
10
NRP1 C-term
50
40
GAPDH
100
70
Notch intracellular domain (positive control for γ-secretase inhibitor

Figure III

A

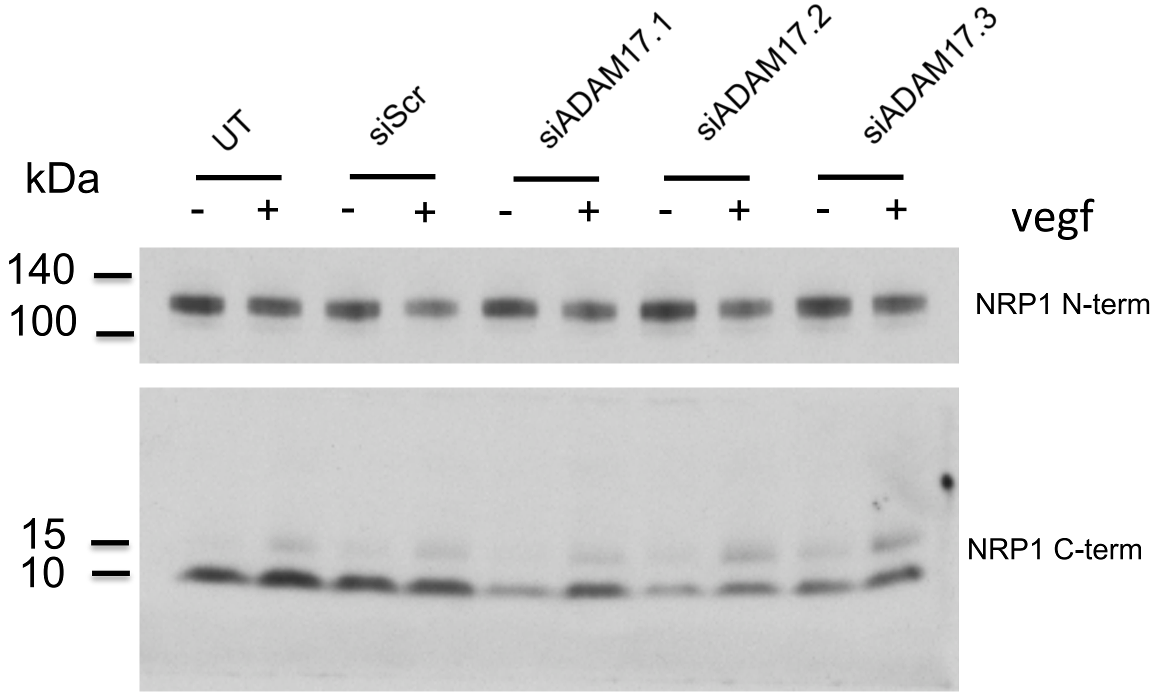

B

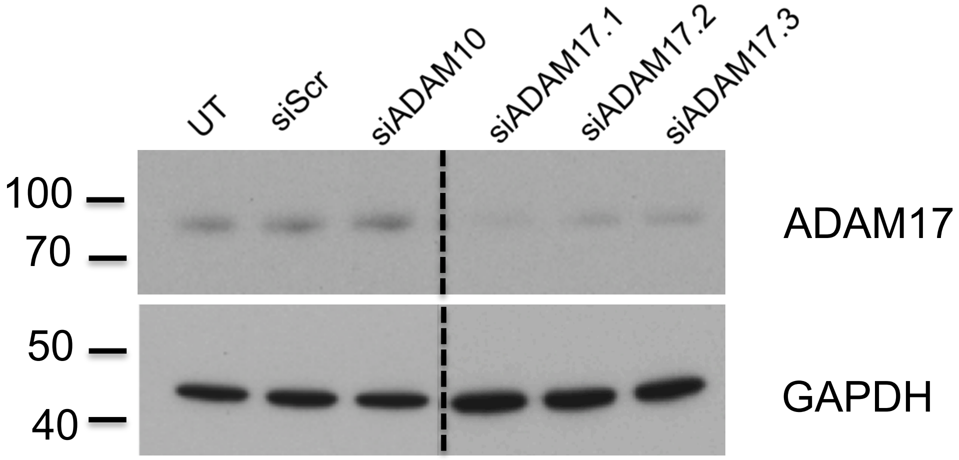

Figure IV

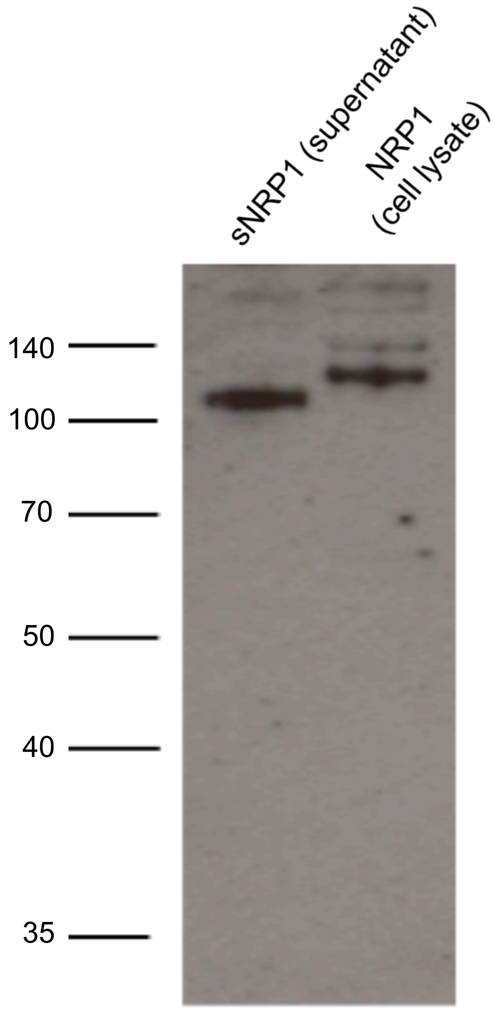

Figure V

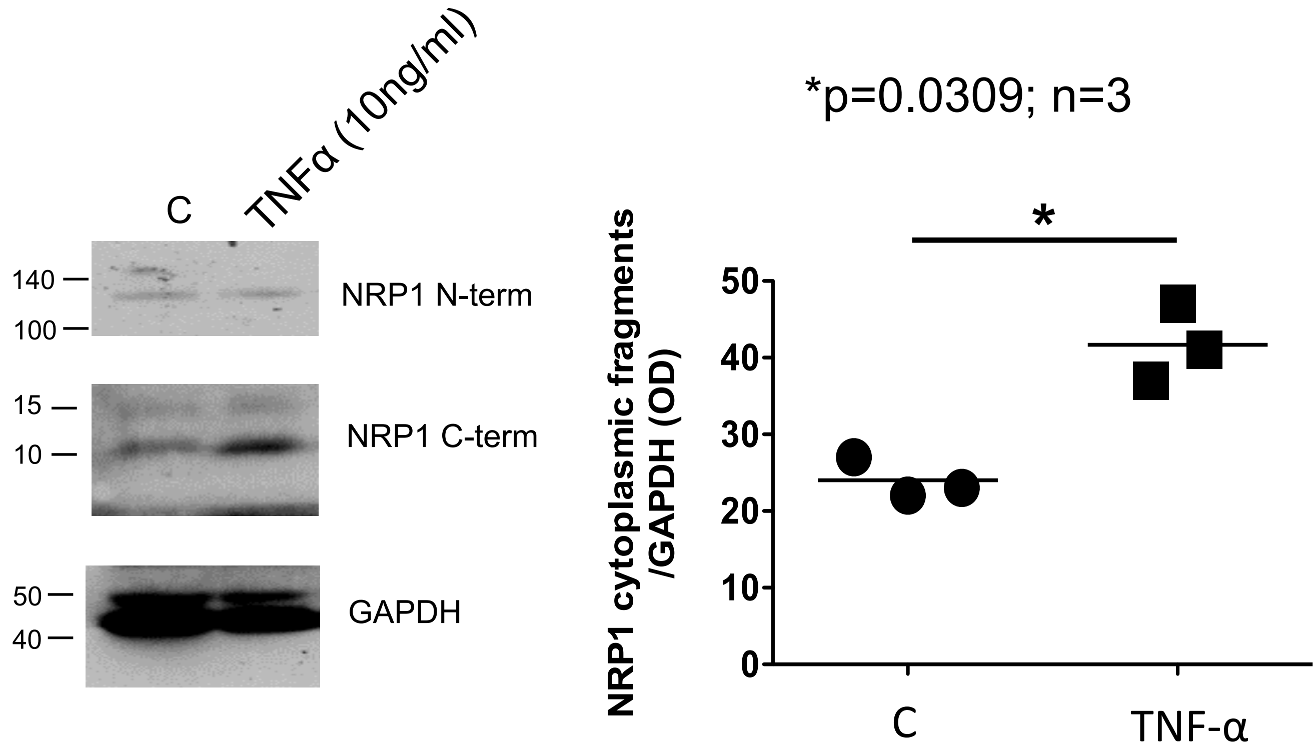
C
TNFα (10ng/ml)
140
100
NRP1 N-term
15
10
NRP1 C-term
50
40
GAPDH
*p=0.0309; n=3
*
NRP1 cytoplasmic fragments /GAPDH (OD)
50
40
30
20
10
0
C
TNF-α

Figure VI

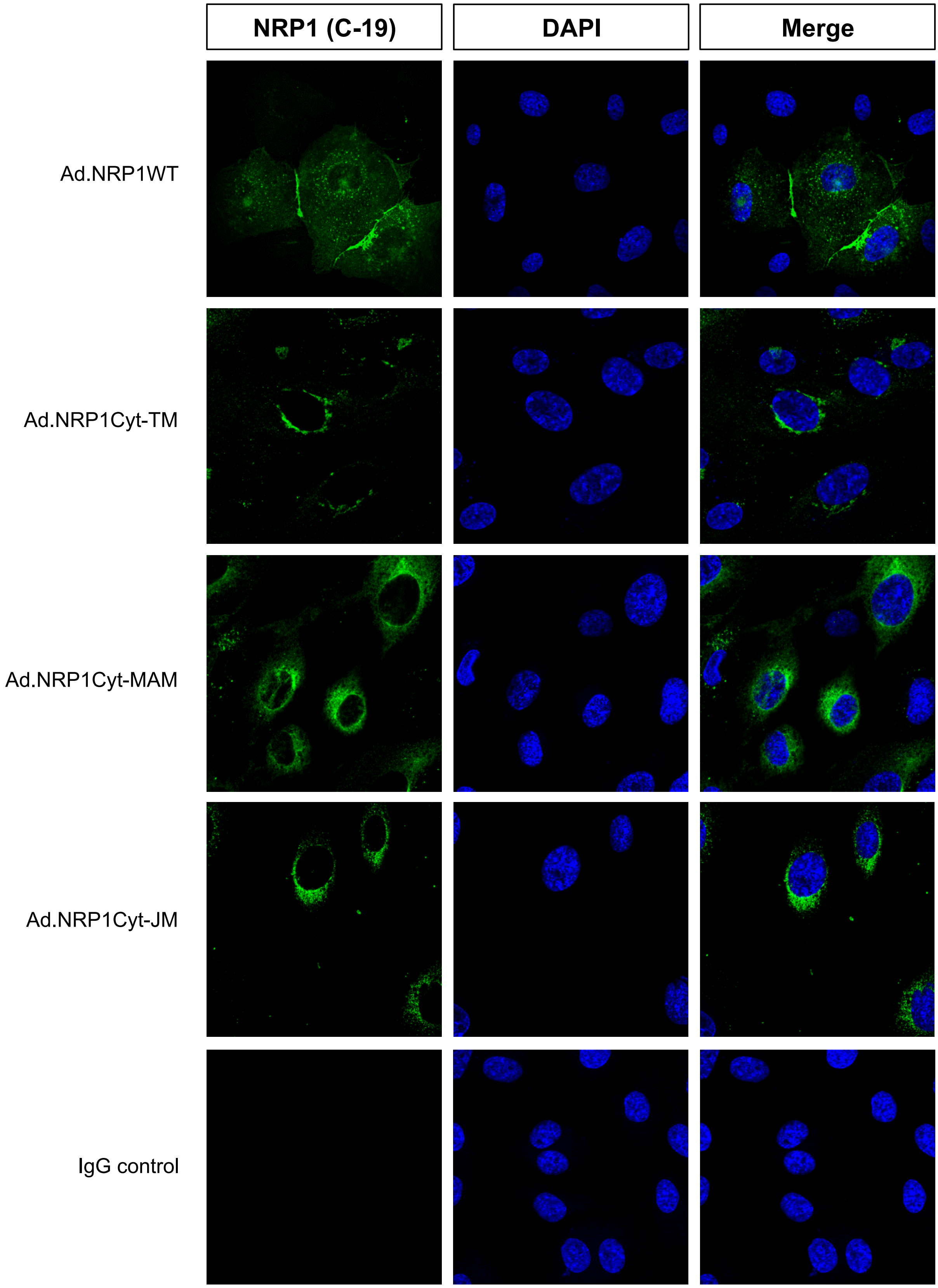
NRP1 (C-19)
DAPI
Merge
Ad.NRP1WT
Ad.NRP1Cyt-TM
Ad.NRP1Cyt-MAM
Ad.NRP1Cyt-JM
IgG control

Figure VII

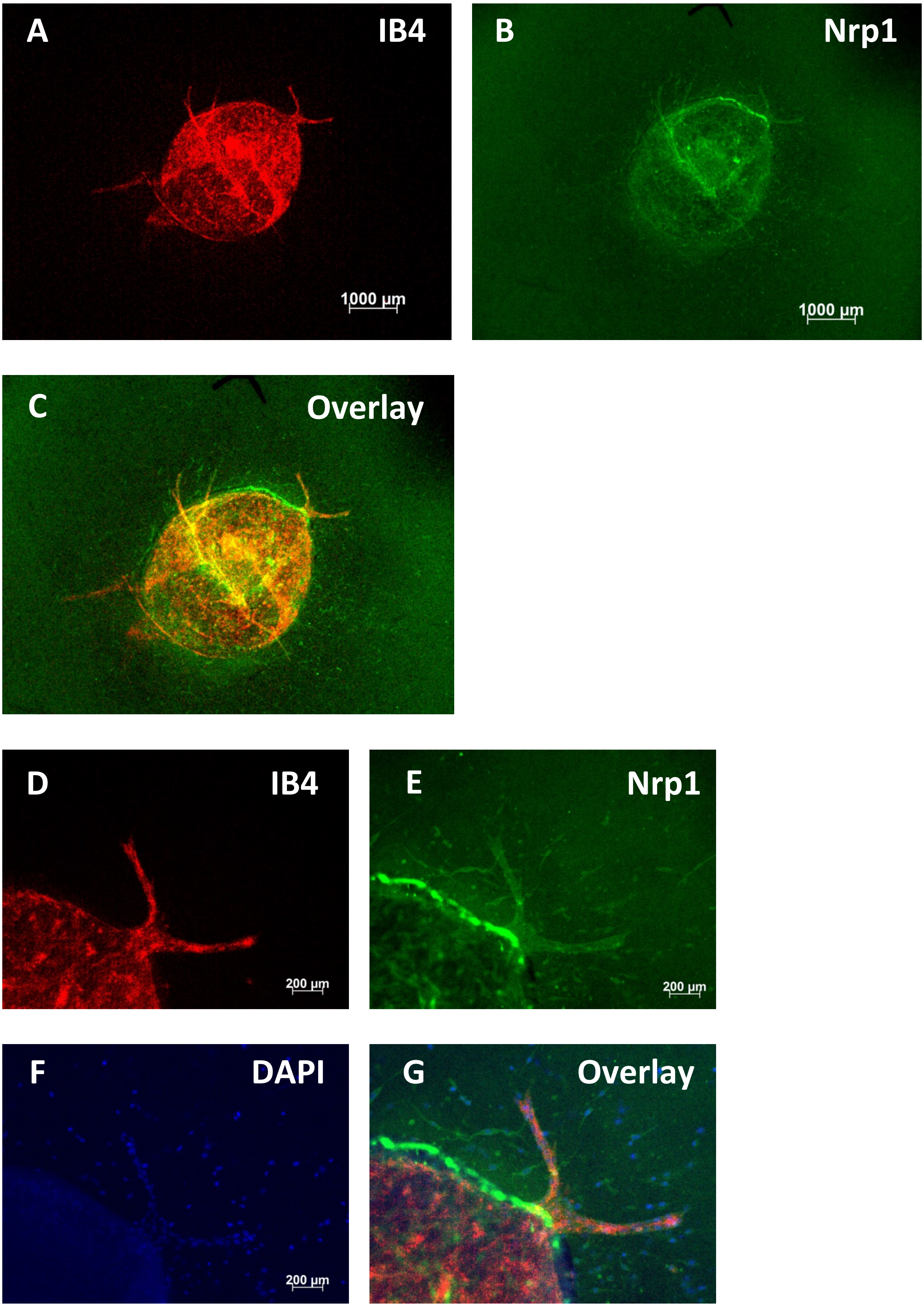
A
IB4
1000 µm
B
Nrp1
1000 µm
C
Overlay
D
IB4
200 µm
E
Nrp1
200 µm
F
DAPI
200 µm
G
Overlay

Figure VIII

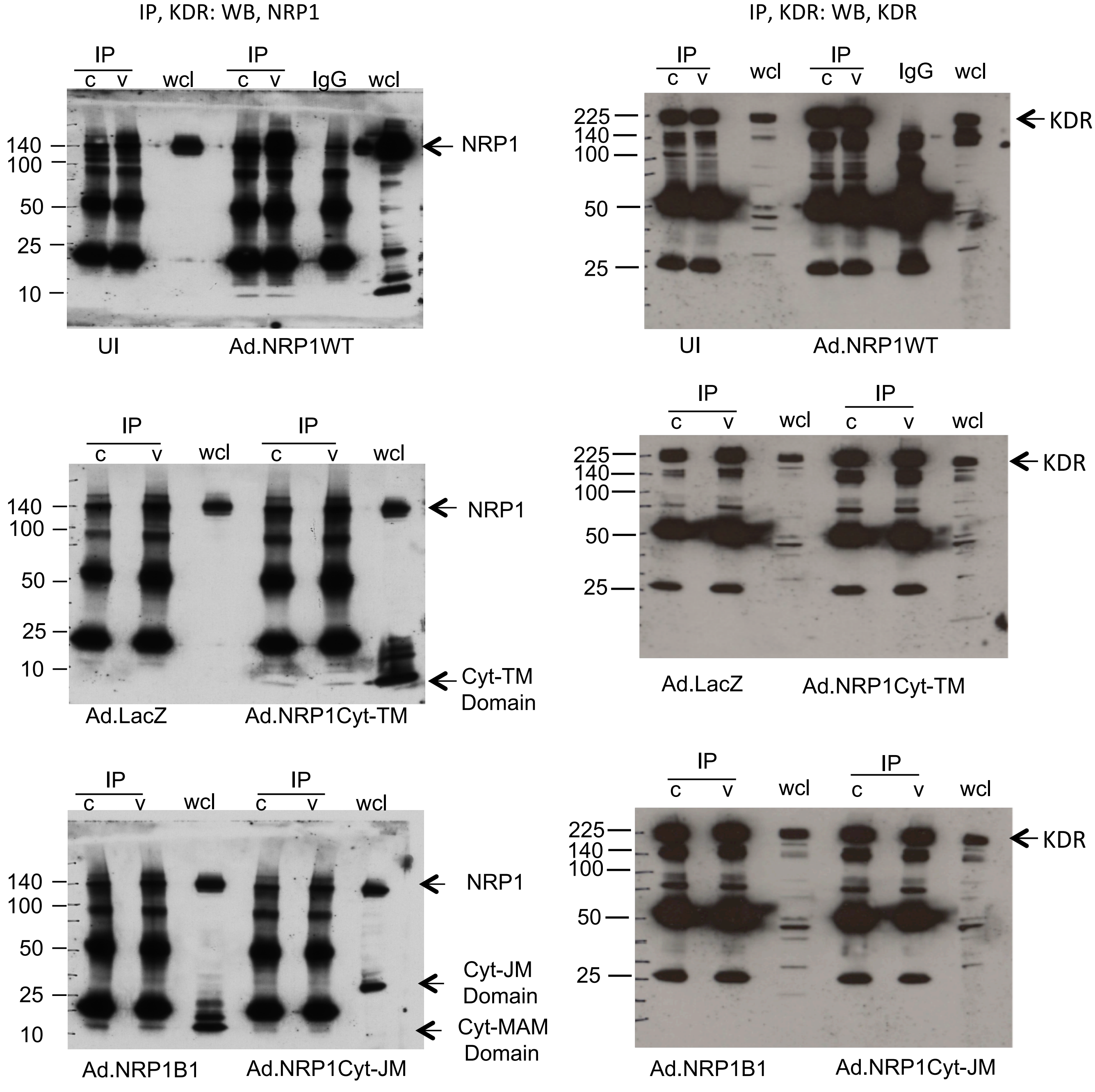
IP, KDR: WB, NRP1
IP
c v
wcl
IP
c v
IgG
wcl
140
100
50
25
10
NRP1
UI
Ad.NRP1WT
IP
c v
wcl
IP
c v
wcl
140
100
50
25
10
NRP1
Cyt-TM
Domain
Ad.LacZ
Ad.NRP1Cyt-TM
IP
c v
wcl
IP
c v
wcl
140
100
50
25
10
NRP1
Cyt-JM
Domain
Cyt-MAM
Domain
Ad.NRP1B1
Ad.NRP1Cyt-JM
IP, KDR: WB, KDR
IP
c v
wcl
IP
c v
IgG
wcl
225
140
100
50
25
KDR
UI
Ad.NRP1WT
IP
c v
wcl
IP
c v
wcl
225
140
100
50
25
KDR
Ad.LacZ
Ad.NRP1Cyt-TM
IP
c v
wcl
IP
c v
wcl
225
140
100
50
25
KDR
Ad.NRP1B1
Ad.NRP1Cyt-JM

Supplementary Figure Legends

Figure I. Human coronary artery smooth muscle cells also express several low (30 kDA and below) molecular weight species recognised by the C-terminal NRP1 antibody.

Figure II. Expression of low molecular weight NRP1 fragments is induced by inhibition of of proteosomal and lysosomal pathways, but not is not affected by inhibition of **γ**-secretase. (**A**) HUVECs overexpressing wild type NRP1 (Ad.NRP1) were incubated with the proteosomal inhibitor, lactacystin at 0-10 µM for 24 h and immunoblotted for full length (AF3870 NRP1 antibody) and low molecular weight (C-19 NRP1 antibody) forms of NRP1. (**B**) HUVECs overexpressing wild type NRP1 (Ad.NRP1) were incubated with the lysosomal inhibitor, chloroquine at 100 µM for 24 h and immnuoblotted for full length (AF3870 NRP1 antibody) and low molecular weight (C-19 NRP1 antibody) forms of NRP1. (**C**) HUVECs were incubated with the **γ**-secretase inhibitors, LY411,575 or DAPT (both at 3 or 10 µM) for 24 h and immunoblotted for full length (AF3870 NRP1 antibody), low molecular weight (C-19 NRP1 antibody) forms of NRP1 and the notch cytoplasmic domain (NICD).

Figure III. Knockdown of ADAM17 has no effect on the expression of low molecular weight NRP1 fragments following VEGF challenge. (**A**) HUVECs were transfected with 3 different siRNAs targeting ADAM17 or a negative control (siScr). After 1 h stimulation with VEGF, lysates were immunoblotted for full length (AF3870 NRP1 antibody), low molecular weight (C-19 NRP1 antibody) forms of NRP1. (**B**) HUVECs were transfected with either siRNA targeting ADAM10, 3 different siRNAs targeting ADAM9, 3 different siRNAs targeting ADAM17 or a negative control (siScr). Knockdown efficacy of ADAM17 siRNA was confirmed by immnublotting with an ADAM17 antibody.

Figure IV. The soluble NRP1 (sNRP1) band generated by ectodomain shedding has greater mobility than the full-length cell-associated NRP1 in SDS-PAGE.

Figure V. Treatment of HUVECs without (C, black circles) or with TNF-α (10ng/ml, black squares) for 24 hours enhances the generation of NRP1 cytoplasmic fragments. *$p<0.05$; Values have been presented as means +/- SEM. Differences between samples were analysed using Student's t-test (between two groups) with the Bonferroni correction for multiple pairwise comparisons after testing for normality and equal variance using Sharpiro-Wilk and Levene tests respectively.

Figure VI. Detection of adenovirally-expressed NRP1 C-terminal constructs by Immunifluorescent staining. HUVECs were transduced adenoviruses overexpressing LacZ, wild type NRP1 (Ad.NRP1WT) or different NRP1 constructs containing the cytoplasmic domain: Ad.NRP1Cyt-TM, Ad.Cyt-JM and Ad.Cyt-MAM. Cells were fixed and immunostained with antibody specific for the NRP1 cytoplasmic domain, as described in materials and methods, and nuclei were counterstained with DAPI.

Figure VII. NRP1 immunofluorescent staining in aortic ring vascular sprouts. Aortic rings were cultured as described in materials and methods and fixed and costained with isolectin B4 (IB4, **A,D**) and an antibody to NRP1 (**B,E**) as described in materials and methods. Localisation of NRP1 in endothelial cells were observed in the overlays (**C,G**). Negative controls showed no staining (data not shown).

Figure VIII. Higher exposures of blots shown in Fig. 7B
